# The role of emotional similarity and emotional accuracy in belonging and stress among first-generation and continuing-generation students

**DOI:** 10.3389/fpsyg.2024.1355526

**Published:** 2024-02-14

**Authors:** Smaranda Ioana Lawrie, Heejung S. Kim

**Affiliations:** ^1^Department of Psychology, Providence College, Providence, RI, United States; ^2^Department of Psychological and Brain Sciences, University of California Santa Barbara, Santa Barbara, CA, United States; ^3^Ewha Womans University, Seoul, Republic of Korea

**Keywords:** emotion, culture, education, belonging, stress, well-being, first-generation students

## Abstract

Extensive research has documented the psychological, social, and academic predicament of first-generation college students. However, basic psychological mechanisms underlying the challenges experienced by these students have been understudied. Taking a cultural psychology perspective, the present research considers the role of emotional (mis)match as a key mechanism for explaining first-generation students’ lowered well-being. A sample of 344 American undergraduate students completed a survey designed to measure two aspects of emotional processing: (1) *Emotional Accuracy* – how accurately students perceive emotional reactions of majority-culture students (continuing-generation junior and senior students who have been socialized into college culture), and (2) *Emotional Similarity* –how similar students’ emotions are to the emotions experienced by majority-culture students. Emotional Accuracy predicted positive outcomes, in general, but was lower among first-generation students. Unexpectedly, Emotional Similarity predicted negative student outcomes. As one of the first studies addressing basic psychological mechanisms in college adjustment, these findings underscore the importance of understanding the roles that specific emotional processes play in social adjustment.

## Introduction

For generations, America prided itself on being the land of opportunity, a place where anyone could live out the *American dream* and rise from rags to riches (Duncan and Murnane, 2011). Today, however, the United States is middle of the pack among other high-income countries in terms of both social inequality and mobility (The World Bank, 2023), which has negative implications for everyone, at both the bottom and top of the social hierarchy. In higher education, a main gateway for upward social mobility, there is a marked gap in academic, social, and psychological well-being and adjustment among college students from minority-culture socioeconomic backgrounds (Wilbur and Roscigno, 2016; Rubin et al., 2019). This study focuses on first-generation students, the first in their families to attend college, who currently make up one-third of the student population at 4-year institutions, and are the quickest growing student demographic (Duncan and Murnane, 2011; Reardon, 2011). Though growing in numbers, first-generation students tend to struggle in college, and the gap in academic outcomes between first- and continuing-generation students (who have at least one parent with a degree from a 4-year institution) is notable. First-generation students typically take fewer credits; receive lower grades; form fewer relationships on campus; suffer from more psychological and physical health problems; and overall, are more likely to drop out and forego their college ambitions entirely (Choy, 2001; Pascarella et al., 2004; Sirin, 2005; Pryor et al., 2007; Kim and Sax, 2009; Rubin, 2012).

Despite extensive research documenting the psychological, social, and academic predicament of first-generation students, basic psychological mechanisms underlying the challenges experienced by these students have been relatively understudied. Filling this gap in the literature, the current research takes a cultural psychology perspective to gain a deeper understanding of psychological reasons for such outcomes. Building on the cultural mismatch theory (CMT; Stephens et al., 2012a), the present research considers the role of emotional (mis-)match as a key mechanism for a sense of belonging and students’ stress levels.

### First-generation students and well-being in college

There are many explanations for the poor outcomes of first-generation students. For instance, many of these students need to balance work and school obligations, live off campus and deal with commutes, come from underperforming high schools, and deal with family guilt from family members who feel left behind (Pascarella et al., 2004; Engle et al., 2006; Covarrubias and Fryberg, 2015; Vasquez-Salgado et al., 2015). Even when these diverse characteristics are taken into account, however, first-generation status remains a negative predictor for college success, suggesting that additional psychological processes may also be at play (Horn and Nuñez, 2000; Choy, 2001, 2002).

CMT proposes that first-generation students experience additional difficulty as they transition to college because they have been socialized in a socioeconomic culture that is different and sometimes at odds with university culture (Stephens et al., 2012a). Typically, having grown up in a more working-class environment, first-generation students bring a more interdependent self to a university environment that heavily emphasizes and values independence. This mismatch causes them to feel out of place in their new environment, somewhat akin to an immigrant in a new country. Chronic concerns about belonging, in turn, have been shown to create increased psychological strain for students, including increased levels of stress (Stephens et al., 2012a,b).

The current research builds on this theory to advance understanding of psychological mechanisms that explain how first-generation students experience this mismatch with their college environment. We propose that part of the answer lies in psychological differences between how first-generation vs. continuing-generation students understand and respond to different emotionally-laden experiences.

### Emotions and social belonging

Individuals may have different emotional responses to seemingly similar situations or stimuli, and this can have important implications for their sense of social belonging. Emotions reflect an individual’s opinions, view of the world, and intentions to act (Frijda et al., 1989; Solomon, 2004). If people experience emotions that are different from those experienced by others around them, they can feel out of place and begin to question their belonging (De Leersnyder et al., 2014).

Research in cultural psychology has found that culture has profound implications on emotional experiences (Mesquita and Janxin, 2007; Tsai and Clobert, 2019). Research has found, for example, that people from different national cultures vary in the intensity and transparency with which they express their emotions (Ekman, 1972; Matsumoto et al., 2008), in the number of emotions that they experience (Mesquita and Karasawa, 2002; Wang, 2004; Kitayama et al., 2006), in the type of emotions that are typically experienced on a daily basis (Mesquita, 2001; Savani et al., 2013), and in the type of emotions that feel particularly good (or bad) (Kitayama et al., 2006; Uchida and Kitayama, 2009).

At the same time, a recent surge of research on social class and college generational status (i.e., first- vs. continuing-generation) suggests that social class cultures have profound implications on psychological functioning in much the same way that national cultures do. Growing up in different social-class contexts fosters and requires different types of behavior; for instance, limited incomes in lower-working-class communities necessitate that people rely on each other more than they would in wealthier communities. Over time, repeated behavioral patterns shape different aspects of the self and patterns of relating to others leading to a more interdependent way of being among lower SES groups and a more independent way of being among higher SES groups (Kraus and Stephens, 2012). Building on and uniting these two distinct lines of research, we reasoned that socialization in different social class environments would also have implications on individuals’ emotional lives. To our knowledge, the link between social class and emotional experiences has not been previously investigated.

### Emotional similarity and emotional accuracy

Research on emotions in social and interpersonal contexts points to several different aspects of emotional processing that could shape psychological outcomes. In the present research, we focus on two aspects of emotional processing: *Emotional Similarity* and *Emotional Accuracy*. These are related but conceptually distinct and could lead to different behavioral and psychological outcomes (Verhofstadt et al., 2008).

*Emotional Similarity* refers to experiencing the same emotions as others in one’s vicinity when in the same situation. Similarity in emotional responses is associated with more rewarding interactions (Locke and Horowitz, 1990), greater empathy (Preston and de Waal, 2002), greater interpersonal coordination (Hatfield et al., 1994; Preston and de Waal, 2002), greater cooperation (Barsade, 2002), increased relationship satisfaction (Anderson et al., 2003; Gonzaga et al., 2007), and decreased stress responses (Townsend et al., 2014).

Extending these findings to intercultural contexts, emotional similarity is indicative of how individuals from one culture feel toward and identify with another culture. Among Korean immigrants, for example, those who have more positive attitudes toward the host culture (i.e., the United States) show greater emotional concordance (i.e., emotional similarity) compared to those who have less positive attitudes (De Leersnyder et al., 2011). Moreover, emotional similarity between an immigrant’s emotional patterns and the emotional patterns typical of the host country’s majority population has been shown to have positive implications for other acculturative processes. Indeed, immigrants who experience more emotional similarity show heightened psychological well-being (De Leersnyder et al., 2015).

The second aspect of emotional processing that we considered in the present research is *Emotional Accuracy*,^1^ which refers to accurate reading and understanding of others’ emotions. A large database of research on emotional intelligence has shown the benefits of being able to read and understand other people’s emotions (Mayer and Salovey, 1997; Mayer et al., 2002). In the school domain, for example, students who score higher in emotional intelligence have numerous positive downstream academic and emotional outcomes (Abdullah et al., 2004). Likewise, research on empathic inferences has found that although there are exceptions, people who are better at mind-reading others’ emotions and thoughts tend to have more positive relationship outcomes (Ickes and Hodges, 2013). Regarding close relationships, research found that individuals who score higher on empathic accuracy are better able to predict and provide the type of support relational partners require (Verhofstadt et al., 2008), and prevent small conflicts from turning into blowouts (Simpson et al., 2001) as well as better align their goals with those of their partner (Berscheid, 1985). Even in short-lived acquaintanceships, individuals higher in emotional accuracy are generally better liked by others (Ahnert et al., 2001).

Aiming to explore the independent roles of emotional accuracy and emotional similarity in explaining the culture clash experienced by first-generation students on a college campus, we conducted a study which measured the two concepts to see how they predict college adjustment.

### The present study

The current research was designed to serve several goals. First, we sought to establish that college generational status influences and shapes emotional responses in similar situations. We hypothesized that first-generation and continuing-generation students would show different patterns of emotions in similar situations and that continuing-generation lower-division students (first- and second-year students) would have emotion profiles more similar to those of continuing-generation upper-division students (juniors and seniors whom we take to represent the “majority” or “host” college culture) compared to first-generation lower-division students.

Secondly, we sought to establish that college generational status influences how well students understand and “read” the emotions of fellow classmates. We hypothesized that compared to first-generation lower-division students, continuing-generation lower-division students would be better at predicting the emotional responses of majority continuing-generation upper-division students.

Lastly, we sought to examine how emotional similarity and emotional accuracy would predict college adjustment outcomes: belonging and stress. We predicted that both a lack of emotional similarity and a lack of emotional accuracy would independently have negative implications for a sense of belonging and stress levels, an important college variable that is related to both psychological well-being and academic outcomes such as GPA (Murff, 2005).

## Methods

### Participants

Participants were 344 undergraduate students at a large and diverse public university in the United States. They were recruited through the Psychology Department’s participant pool and received course credit. The study was evaluated and approved by an IRB committee at the sponsoring university.

Two hundred and fifty-two lower-division students completed the study. Of these, 60.7% were first-year students (*N* = 153), and 39.3% were second-year students (*N* = 99) (Age *M* = 18.47, *SD* = 0.66; 71% female). Parental education was used to distinguish between first- and continuing-generation students. Students who had at least one parent with a bachelor’s degree or more were coded as “continuing-generation.” All other students were considered “first-generation” following criteria used in the past (Stephens et al., 2014). Our sample consisted of *n* = 111 first-generation (48.6% Latino/a-Americans, 28.8% Asian Americans, 9% European Americans, 2.7% African Americans & 10.8% other; 73.9% females) and *n* = 141 continuing-generation lower-division students (10.6% Latino/a-Americans, 39% Asian Americans, 39% European Americans, 2.8% African Americans & 8.6% other; 68.8% females).

An additional 92 continuing-generation upper-division (i.e., juniors and seniors) respondents completed the study to be used in emotional similarity and emotional accuracy calculations for computing “host” or “majority” culture averages because they have had sufficient time to acculturate to college culture. Of these, 79.3% were juniors (*N* = 73), and 20.7% were seniors (*N* = 19) (Age *M* = 21.16, *SD* = 2.24; 59.8% European Americans, 16.3% Asian Americans, 6.5% Latino/a-Americans, 4.3% African Americans, and 13.1% other; 65.2% females).

### Procedure

Participants came to a lab and individually completed an anonymous online survey in Qualtrics. This study was part of a larger study looking at college adjustment among first- vs. continuing generation students. After completing the survey, participants were fully debriefed. Materials are posted online at: https://osf.io/jvqpw/?view_only=fc759f8e2e274416ae70afefef61220d.

### Measures

#### Social belonging

Social belonging was measured with an eight-item subset of the Sense of Social and Academic Fit Scale (Walton and Cohen, 2007). Previous research (Lawrie et al., 2023) has found that academic and social belonging are two distinct constructs with distinct implications for student outcomes. In line with the CMT, we wanted to focus on social belonging, so we used only items related to social belonging. Participants were asked to indicate on a 7-point Likert scale whether they agree with certain statements (1 = *Strongly disagree* to 7 = *Strongly agree*, e.g., *“People at [university name] accept me*;” *“I feel like an outsider at [university name].”* Reliability was good (ɷ = 0.93, ⍺ = 0.92). Two items were reverse coded.

#### Stress

Stress was measured using the ten-item Perceived Stress Scale (Cohen et al., 1994). Participants were asked how often they felt or thought a certain way in the past month on a seven-point Likert Scale (1 = *Never* to 7 = *Very often*, e.g., “*In the last month, how often have you felt that you were unable to control the important things in your life*,” *“In the last month, how often have you been upset because of something that happened unexpectedly?”*). Reliability was good (ɷ = 0.86, ⍺ = 0.86). Four items were reverse coded.

#### Emotional similarity and emotional accuracy

Emotional similarity and emotional accuracy between first- and second-year students and the majority culture students was measured using the Emotional Patterns Questionnaire (EPQ) (De Leersnyder et al., 2011). Although the EPQ was initially developed as a measure of immigrants’ emotional similarity to their host group (e.g., Korean immigrants in the USA), the questions are general and applicable to any populations. Thus, instructions and emotion words used in the current study were identical to previous research. However, given our sample consisted solely of university students, we focused exclusively on the school context and did not include prompts related to work or family life. Also, for brevity, only two negative prompts were used as past research has found greater emotional variability when participants were promoted to think about negative compared to positive situations (De Leersnyder et al., 2011, 2020). In the Negative Engaged prompt, students were asked to write about *“an occasion at school in which they felt bad about their relationship with others*,” and in the Negative Disengaged prompt, students were asked to write about “*an occasion at school in which they felt bad about things that happened to them personally.*” After writing about each prompt, participants rated themselves on 30 emotions elicited by the prompt (e.g., proud, angry, guilty, friendly) using a 7-point Likert-type scale (1 = *not at all* to 7 = *extremely*). Items were chosen to represent emotions that vary in valence, arousal, and social engagement dimensions (i.e., engaged emotions which have to do with a relationship or disengaged emotions that have to do with the independent self) (Barrett and Russell, 1998; Kitayama et al., 2006).

To calculate emotional similarity, we computed the average scores of continuing-generation upper-level students for each of the 30 emotions measured in each of 2 prompts and correlated these scores to lower-division students’ individual responses on these same items. Thus, each lower-division participant’s individual emotional pattern (based on their ratings of emotions) was correlated to the average majority culture’s emotional pattern, yielding two scores–one for the Negative Disengaged prompt and the other for the Negative Engaged prompt. These correlations represent participants’ emotional similarity score - that is, the similarity between a participants’ unique emotional pattern and the mean pattern of the larger college culture (continuing-generation culture). All scores were converted to Fisher’s Z-scores to ensure a normal distribution, ranging between −3 and + 3 (see De Leersnyder et al., 2011 for additional information on score calculations).

After rating their own emotions in each scenario, students were subsequently asked to rate how they thought the “*typical [university name] student*” would respond in the same situation. In other words, they were asked to infer the emotional responses of majority-culture students. These responses were then correlated to the actual averages of the continuing-generation upper-level students in the same way that Emotional Similarity scores were computed. Scores were, again, converted to Fisher’s Z-scores.

### Analytic approach

As a first step, we ran *T*-tests to establish differences between first- and continuing- generation students on all study variables. Zero-order correlations were then analyzed to get a better understanding of the relationships between study variables. To test the role of emotional accuracy/similarity in predicting college adjustment outcomes, a multi-group Structural Equation Modeling (SEM) was used. Finally, we tested whether our model was invariant across generational status.

## Results

To first determine any differences between first- and continuing-generation students on key study variables, *T*-tests were employed (see Table 1). Contrary to our first hypothesis, there were no significant differences in Negative Engaged Emotional Similarity (NES) (*t*(209.77) = −1.43, *p* = 0.15) or Negative Disengaged Emotional Similarity (NDS) (*t*(250) = 0.36, *p* = 0.72) between first-generation and continuing-generation students.

**Table 1:** Descriptive statistics of key variables, split by college generational status.

	First-generation students			Continuing-generation students			*t*-value	*df*	*p*
	*M*	*SD*	Range (Min; Max)	*M*	*SD*	Range (Min; Max)			
NES	0.45	0.49	−0.79; 1.46	0.53	0.40	−0.85; 1.29	−1.43	209.77	0.15
NDS	0.72	0.42	−0.61; 1.47	0.70	0.45	−0.87; 1.54	0.36	250	0.72
NEA	0.45	0.45	−0.83; 1.32	0.60	0.42	−0.76; 1.31	−2.60	250	. 01
NDA	0.74	0.47	−1.07; 1.62	0.74	0.39	−0.63; 1.54	0.16	250	0.88
Belonging	5.07	1.09	2.75; 7.00	5.57	1.08	1.25; 7.00	−3.61	250	0.000
Stress	4.12	0.98	1.20; 7.00	4.06	1.03	1.70; 7.00	0.42	250	0.68

NES, Negative Engaged Emotional Similarity; NDS, Negative Disengaged Emotional Similarity; NEA, Negative Engaged Emotional Accuracy; NDA, Negative Disengaged Emotional Accuracy. Possible scores range from −3 to +3 for first four items and 1 to 7 for last two items.

Contrary to our second hypothesis, there was no significant difference in Negative Disengaged Emotional Accuracy (NDA) (*t*(250) = 0.16, *p* = 0.88) between first-generation and continuing-generation students. However, there was a significant difference in Negative Engaged Emotional Accuracy (NEA) (*t*(250) = −2.60, *p* = 0.01); continuing-generations students showed higher Accuracy.

In terms of outcome variables, there were no observable differences on Stress (*t*(250) = 0.42, *p* = 0.68), but Belonging was significantly lower (*t*(250) = −3.61, *p* < 0.01) for first-generation students. See Table 2 for descriptive statistics for emotion ratings.

**Table 2:** Mean emotions across different types of emotional situations.

	Emotion scale																							
	Positive engaged						Positive disengaged						Negative engaged						Negative disengaged					
	First gen		Cont gen		Upper-level students		First gen		Cont gen		Upper-level students		First gen		Cont gen		Upper-level students		First gen		Cont gen		Upper-level students	
	M	SD	M	SD	M	SD	M	SD	M	SD	M	SD	M	SD	M	SD	M	SD	M	SD	M	SD	M	SD
NE	2.70**	1.51	2.22	1.04	2.29	1.25	2.25	1.11	2.05	0.96	2.09	1.01	**3.29**	**1.30**	**3.22**	**1.08**	**3.38**	**1.26**	3.35	1.28	3.36	1.20	3.68	1.33
NE-P	2.62**	1.40	2.06	1.13	–	–	2.22*	1.10	1.94	0.91	–	–	**3.18**	**1.23**	**3.21**	**1.17**	–	–	3.27	1.26	3.41	1.31	**–**	**–**
ND	2.11*	1.29	1.77	0.91	2.06	1.10	1.83	0.94	1.76	0.83	1.86	0.95	3.28	1.38	3.13	1.19	3.70	1.34	4.10*	**1.37**	**3.74**	**1.30**	**3.99**	**1.35**
ND-P	2.01*	1.29	1.65	0.87	–	–	1.83	1.00	1.64	0.74	–	–	3.29	1.42	3.05	1.15	**–**	**–**	4.02*	**1.41**	**3.66**	**1.30**	**–**	**–**

Means of emotional experiences on a scale from 1 (not at all) to 7 (a great deal). Bold emotion scales match the prompt. Superscripts signal significant differences between first- and continuing-generation. NE, Negative Engaged Self-Reported; NE-P, Negative Engaged Majority Perceived; ND, Negative Disengaged Self-Reported; ND-P, Negative Disengaged Majority Perceived.

*p < 0.05; **p < 0.01.

Next, zero-order correlations were analyzed to get a better understanding of relationships between study variables before moving onto the Structural Equation Modeling (SEM) framework. Emotional Similarity and Emotional Accuracy scores were significantly correlated, but correlations were not high enough to suggest multicollinearity. See Table 3.

**Table 3:** Correlations, split by college generational status.

	NES	NEA	NDS	NDA	Social belonging	Stress
NES	1	0.70**	0.35**	0.26**	−0.14	0.35**
NEA	0.45**	1	0.30**	0.40**	0.04	0.20*
NDS	0.22*	0.15	1	0.75**	−0.16	0.40**
NDA	0.01	0.21*	0.73**	1	−0.05	0.26**
Social belonging	−0.10	0.06	−0.06	0.03	1	−0.38^**^
Stress	0.25^**^	−0.02	0.28^**^	0.01	−0.18	1

NES, Negative Engaged Emotional Similarity; NDS, Negative Disengaged Emotional Similarity, NEA – Negative Engaged Emotional Accuracy, NDA – Negative Disengaged Emotional Accuracy.

Numbers below diagonal are first-gen. students, above diagonal are continuing-gen. students.

*p < 0.05; **p < 0.01.

Finally, we used a SEM framework to test whether Social Belonging mediates the relationship between Emotional Similarity and Emotional Accuracy and Stress. IBM’s SPSS (Version 24) and Amos (Version 20), with maximum likelihood estimation (Arbuckle, 2011), were used. A constellation of model fit indices were analyzed to ascertain model fit. These included the chi-square test, the room mean square of approximation (RMSEA), the comparative fit index (CFI), the standardized root-mean square residual (SRMR), and the Bentler and Bonett (1980) Normed Fit Index (NFI). For NFI, a value of over 0.9 indicates good model fit, while RMSEA (Steiger, 1990) should show values of under 0.08 to indicate good model fit (Cangur and Ercan, 2015). SRMR indicates an acceptable fit when it produces a value smaller than 0.10 (Cangur and Ercan, 2015), while CFI shows acceptable fit when its value is over 0.90 (Kline, 2005).

We also tested for the moderating effect of student generational status - that is whether the same pattern of relationships is present among first- and continuing- generation students. The overall model structure for the amended model is almost identical for first- and continuing-generation students (See Supplementary materials).

**Figure 1:**
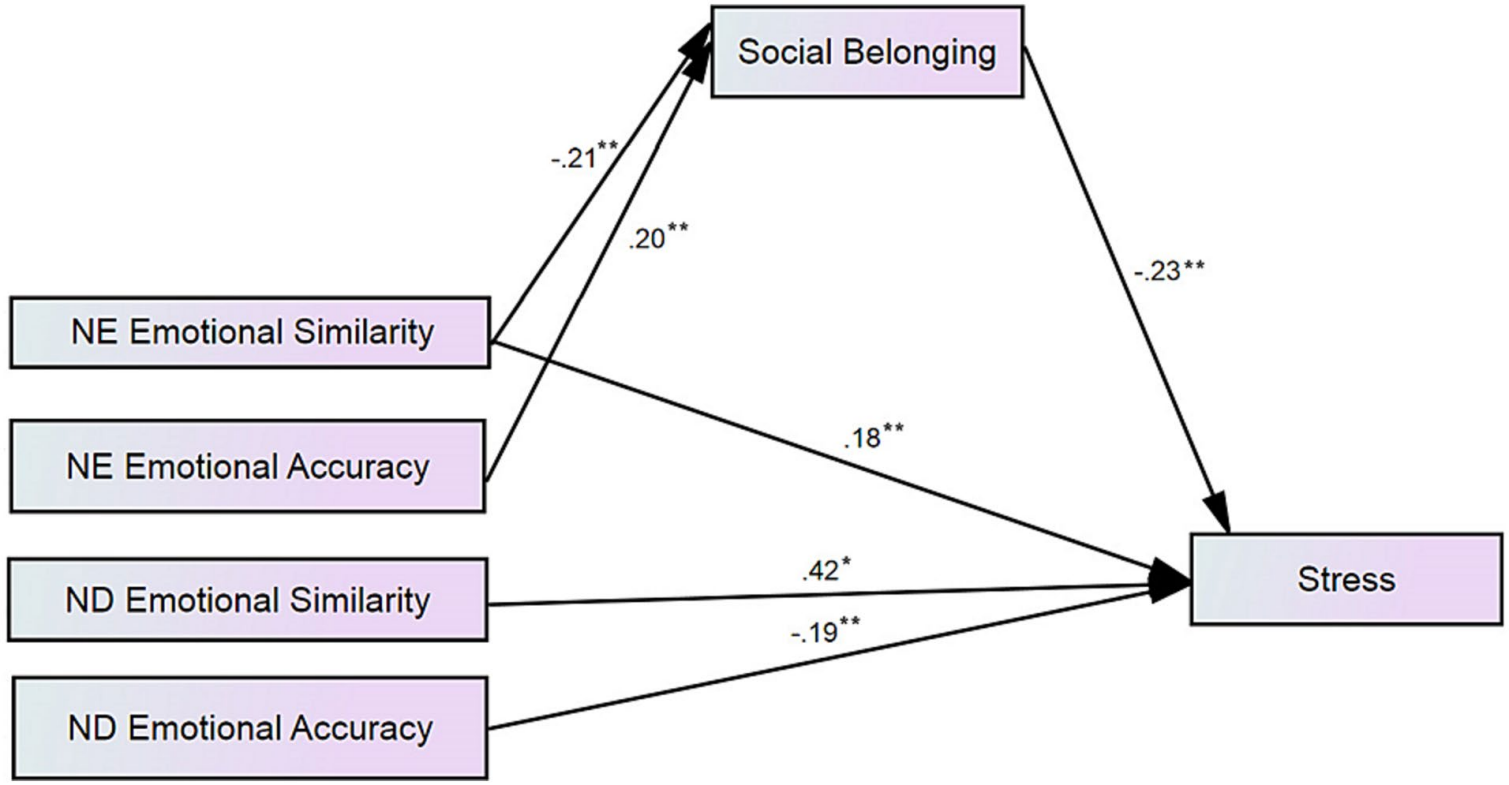
Results of the structural equation model (SEM) used to test the mediation of belonging on stress for both types of students. *p < 0.01, **p < 0.001.

After removing the direct paths that were non-significant, the modified overall model showed great fit (*χ*^2^ = 3.93, df = 3, *p* = 0.27; CFI = 0.99; NFI = 0.99 SRMR = 0.01; RMSEA = 0.03 CI 10% [0.00, 0.11]) (see Figure 1). Belonging mediates the relationship between Negative Engaged Emotional Similarity and Stress, as well as between Negative Engaged Emotional Accuracy and Stress. However, Belonging does not mediate the relationship between Negative Disengaged Emotional Similarity and Stress or Negative Disengaged Emotional Accuracy and Stress. This is not entirely surprising given that the engaged prompt had students write about a situation involving others and belonging is a relational measure, whereas the disengaged prompt had students write about a situation that did not involve others.

What stands out is that, overall, Accuracy is associated with better outcomes than Similarity. Negative Engaged Emotional Accuracy positively predicted Belonging (*B* = 0.51 (0.22), 95% CI [0.02, 0.96], *p* < 0.01, β = 0.20 (0.09), 95% CI [−0.01, 0.35], *p* < 0.05) whereas unexpectedly, Negative Engaged Emotional Similarity negatively predicted Belonging (*B* = −0.53 (0.23), 95% CI [−0.97, 0.00], *p* < 0.01, β = −0.21 (0.09), 95% CI [−0.37, 0.01], *p* < 0.05). Likewise, Negative Disengaged Emotional Similarity significantly positively predicted Stress (*B* = 0.95 (0.17), 95% CI [0.55, 1.26], *p* < 0.001, β = 0.42 (0.08), 95% CI [0.26, 0.56], *p* < 0.05), whereas Negative Disengaged Emotional Accuracy negatively predicted Stress (*B* = −0.45 (0.17), 95% CI [−0.78, −0.10], *p* < 0.05, β = −0.19 (0.07), 95% CI [−0.33, −0.04], *p* < 0.05). Negative Engaged Emotional Similarity also positively predicted Stress (*B* = 0.41 (0.15), 95% CI [0.11, 0.73], *p* < 0.05, β = 0.42 (0.08), 95% CI [−0.01, 0.33], *p* < 0.05).

We also tested the direct-only (non-mediation) effects of Emotional Similarity and Emotional Accuracy on Stress followed by the indirect-only mediation effects of Emotional Similarity and Emotional Accuracy through Belonging in the SEM context. Mirroring results above, there was a significant positive direct effect of Negative Engaged Emotional Similarity on Stress (*B* = 0.41 (0.15), 95% CI [0.11, 0.73], *p* < 0.05, β = 0.18 (0.07), 95% CI [0.04, 0.38], *p* < 0.05). This was also true for the direct effect of Negative Disengaged Emotional Similarity on Stress (*B* = 0.95 (0.17), 95% CI [0.55, 1.26], *p* < 0.05, β = 0.42 (0.07), 95% CI [0.26, 0.56], *p* < 0.05). However, there was a significant negative direct effect of Negative Disengaged Emotional Accuracy on Stress (*B* = −0.45 (0.18), 95% CI [−0.78, −0.10], *p* < 0.05, β = −0.19 (0.07), 95% CI [−0.33, −0.04], *p* < 0.05).

Significant indirect effects of Negative Engaged Emotional Similarity on Stress through Belonging were observed (*B* = 0.11 (0.05), 95% CI [0.02, 0.24], *p* < 0.05, β = 0.05 (0.02), 95% CI [0.01, 0.11], *p* < 0.05). Belonging fully mediates the relationship between Negative Engaged Emotional Similarity and Stress, such that higher Emotional Similarity predicts less Belonging which in turn predicts higher Stress. Significant indirect effects of Negative Engaged Emotional Accuracy on Stress through Belonging were also found (*B* = −0.11 (0.05), 95% CI [−0.22, −0.01], *p* < 0.05, β = −0.05 (0.02), 95% CI [−0.11, −0.01], *p* < 0.05) such that higher Accuracy predicts higher Belonging which in turn predicts lower Stress.

To summarize, Emotional Similarity had negative implications on Stress both directly and through the mediating role of Belonging. Conversely, Emotional Accuracy had positive implications on Stress directly and through the mediating role of Belonging. With the exception of Belonging (where Belonging predicted Stress only for continuing generation students), no differences were observed across generational status.

## Discussion

The current research was designed to test three main hypotheses. First, we hypothesized that first-generation students would show less similar patterns of emotions to the “majority culture” students (continuing-generation upper-division students) compared to continuing-generation students. Secondly, we hypothesized that compared to first-generation students, continuing-generation students would be better at predicting the emotional responses of majority continuing-generation upper-division students. Lastly, we hypothesized that a lack of emotional similarity and a lack of emotional accuracy would independently have negative implications for a sense of belonging and stress.

Our hypotheses were partially supported. Contrary to the first hypothesis, we found no significant differences between first-generation and continuing-generation students in emotional similarity. That is, although there were some mean-level differences in specific emotional experiences between the two groups (e.g., first-generation students’ overall experience and perceive more positive engaged emotions than continuing-generation students), their emotional profiles did not differ. Our second hypothesis was partially supported; we found a significant difference between the two groups in emotional accuracy in the negative engaged prompt. As expected, continuing-generation lower-division students scored higher in emotional accuracy compared to first-generation students. At least for the engaged prompt, continuing-generation lower-division students were better able to infer the emotional responses of majority-culture students whereas first-generation students seemed to have a more difficult time inferring the emotions of majority-culture students. This finding makes sense given that the two groups of students were most likely socialized in quite different socioeconomic (SES) cultures.

The surprising finding was that emotional similarity, independent of accuracy, predicted negative student outcomes. This result counters existing findings in the literature on the psychological benefits of emotional similarity, especially when we looked at its role independent from that of emotional accuracy. This unexpected result may be explained by the fact that the prompts focused solely on negative situations, whereas past studies using the EPQ included both positive and negative prompts. Given that the typical emotional profiles are characterized by relatively high negative emotions and low positive emotions, those who are emotionally *dis*similar should show emotional profiles with relatively high positive emotions and/or relatively low negative emotions. Thus, it is possible that students who experience not only negative emotions, but also some positive emotions that offset negative consequences of experiencing predominantly negative emotions. Given that emotional similarity, predicting worse outcomes, including more stress, makes sense.

In contrast, emotional accuracy predicted positive outcomes, supporting the hypothesis. Broadly speaking, increased emotional accuracy was both directly and indirectly associated with decreased stress for first- and continuing-generation students. Emotional accuracy is essentially cognitive empathy/perspective taking, resulting in accuracy without necessarily experiencing the emotions of others (Verhofstadt et al., 2008). This distinction may help explain the divergent patterns of results in the current study. Most previous research in cultural psychology has focused exclusively on emotional similarity, but the current study suggests that an important future direction for the field is to further investigate the role of emotional accuracy, including with samples from different national cultures.

When both factors are considered simultaneously, emotional accuracy provides stronger psychological benefits for students than emotional similarity; however, similarity and accuracy are related factors, and similarity would increase accuracy, as experiencing the same emotions as another individual negates the need for perspective taking. Alternatively, accuracy could increase similarity, as emotional accuracy reflects a form of perspective taking. It is therefore not surprising that those two factors are fairly strongly correlated with each other, although there are meaningful differences between them, one being that experiencing similar emotions as others may or may not involve perspective taking, whereas accurately assessing others’ emotions requires accurately inferring others’ feelings (Ickes, 1993). Given this distinction, it is possible that emotional similarity, in our analysis, may be capturing emotional similarity without perspective taking, and this may be yet another reason for the lack of positive outcomes related to similarity.

Like all research, the current study is not without limitations. First, the study is cross-sectional, so a causal link between emotional similarity/accuracy and college adjustment outcomes cannot be established. As the present study provided the initial evidence of the relationships, future research should use different methodology, such as a longitudinal study or an experiment (e.g., increasing emotional accuracy by providing factual information on emotions of others) to understand causality of the association. Second, reflecting the gender imbalance present in the psychology major, our sample is made up of a larger proportion of females compared to males. While this issue points to the need to test the generalizability of the findings, at least, the gender breakdown was comparable across first- and continuing-generation groups, and thus, unlikely to be a confound. More importantly, the ethnic breakdown of each group differed a great deal, reflecting the societal reality in which underrepresented ethnic minorities have lower SES status. It is possible that some of the generation differences found are due to ethnic cultural difference, in addition to SES difference. At the same time, it is important to note that the role of emotional similarity/accuracy in predicting college adjustment outcomes did not differ between first- and continuing generation students.

## Conclusion

There are several possible conclusions that can be drawn from this research. A possible significant conclusion is that accuracy is lower for first-generation students, and this may have implications for outcomes in college. The upside is that students can be taught a better understanding of majority-culture emotions, thereby increasing their accuracy (Ashkanasy and Dasborough, 2003; Pool and Qualter, 2012), thus providing space for the development of a potentially useful intervention that could supplement other interventions designed to help first-generation students and other minority student groups to succeed in college. This research makes an important contribution to the field, because, as far as we know, it is one of the first studies to show that socioeconomic cultures, like national cultures, shape individuals’ emotional lives, thereby contributing to the newer frontiers of cultural psychology which tackles other forms of culture in addition to national cultures. More generally, the findings underscore the value in considering emotion processes in advancing the understanding of why and how individuals form social relationships and identities.

## Data availability statement

The datasets presented in this study can be found in online repositories. The names of the repository/repositories and accession number(s) can be found at: https://osf.io/jvqpw/?view_only=fc759f8e2e274416ae70afefef61220d.

## Ethics statement

The studies involving humans were approved by UCSB Institutional Review Board. The studies were conducted in accordance with the local legislation and institutional requirements. The participants provided their written informed consent to participate in this study.

## Author contributions

SL: Conceptualization, Methodology, Writing – original draft, Writing – review & editing, Data curation, Formal analysis, Project administration. HK: Conceptualization, Methodology, Writing – review & editing, Funding acquisition, Supervision.

## Footnotes

^1^The term empathic accuracy is typically used, but we used “emotional accuracy” to highlight the present focus on accuracy in anticipating specifically others’ emotions.

## References

[ref1] Abdullah M. C. Elias H. Mahyuddin R. Uli J. (2004). Emotional intelligence and academic achievement among Malaysian secondary students. Pak. J. Psychol. Res. 9, 105–121.

[ref2] Ahnert R. Klein K. Veach D. Hodges S. (2001). “Understanding empathic accuracy” in Poster presented at the meeting of the Society of Personality and Social Psychology (San Antonio, TX)

[ref3] Anderson C. Keltner D. John O. P. (2003). Emotional convergence between people over time. J. Pers. Soc. Psychol. 84, 1054–1068. doi: 10.1037/0022-3514.84.5.1054 12757148

[ref4] Arbuckle J. (2011). IBM SPSS Amos 20 user’s guide Amos Development Corporation.

[ref5] Ashkanasy N. M. Dasborough M. T. (2003). Emotional awareness and emotional intelligence in leadership teaching. J. Educ. Bus. 79, 18–22. doi: 10.1080/08832320309599082

[ref6] Barrett L. F. Russell J. A. (1998). Independence and bipolarity in the structure of current affect. J. Pers. Soc. Psychol. 74, 967–984. doi: 10.1037/0022-3514.74.4.967

[ref7] Barsade S. G. (2002). The ripple effects: emotional contagion and its influence on group behavior. Adm. Sci. Q. 47, 644–675. doi: 10.2307/3094912

[ref9001] Bentler P. M. Bonett D. G. (1980). Significance tests and goodness of fit in the analysis of covariance structures. Psychological Bulletin, 88, 588–606. doi: 10.1037/0033-2909.88.3.588

[ref8] Berscheid E. (1985). “Interpersonal attraction” in The handbook of social psychology. eds. Lindzeyand G. Aronson E. (New York City, NY: Random House).

[ref9002] Cangur S. Ercan I. (2015).Comparison of model fit indices used in structural equation modeling under multivariate normality. Journal of Modern Applied Statistical Methods. 14, 152–167. doi: 10.22237/jmasm/1430453580

[ref9] Choy S. (2001). Students whose parents did not go to college: Postsecondary access, persistence, and attainment: findings from the condition of education. Washington, DC: National Center for Education Statistics, U.S. Department of Education.

[ref10] Choy S. P. (2002). Access & persistence: Findings from 10 years of longitudinal research on students. Washington, DC: American Council on Education, Center for Policy Analysis.

[ref11] Cohen S. Kamarck T. Mermelstein R. (1994). Perceived stress scale. Meas. Stress Guide Health Soc. Scient. 10, 1–2.

[ref12] Covarrubias R. Fryberg S. A. (2015). Movin’on up (to college): first-generation college students’ experiences with family achievement guilt. Cult. Divers. Ethn. Minor. Psychol. 21, 420–429. doi: 10.1037/a0037844 25198416

[ref13] De Leersnyder J. Kim H. Mesquita B. (2015). Feeling right is feeling good: psychological well-being and emotional fit with culture in autonomy-versus relatedness-promoting situations. Front. Psychol. 6:630. doi: 10.3389/fpsyg.2015.00630 26042063 PMC4436561

[ref14] De Leersnyder J. Kim H. S. Mesquita B. (2020). My emotions belong here and there: extending the phenomenon of emotional acculturation to heritage culture fit. Cognit. Emot. 34, 1573–1590. doi: 10.1080/02699931.2020.1781063 32552290

[ref15] De Leersnyder J. Mesquita B. Kim H. S. (2011). Where do my emotions belong? A study of immigrants’ emotional acculturation. Personal. Soc. Psychol. Bull. 34, 451–463. 10.1177/0146167211399103 21357754

[ref16] De Leersnyder J. Mesquita B. Kim H. Eom K. Choi H. (2014). Emotional fit with culture: a predictor of individual differences in relational well-being. Emotion 14, 241–245. doi: 10.1037/a0035296 24364853

[ref17] Duncan G. J. Murnane R. J. (2011). “Introduction: the American dream, then and now” in Whither opportunity?: Rising inequality, schools, and children’s life chances. eds. Duncan G. J. Murnane R. J. (New York, NY: Russell Sage Foundation).

[ref18] Ekman P. (1972). “Universals and cultural differences in facial expression of emotion” in Nebraska symposium on motivation. ed. Cole J. (Lincoln, NE: University of Nebraska Press).

[ref19] Engle J. Bermeo A. O'Brien C. (2006). Straight from the source: what works for first-generation college students. Pell Institute for the Study of Opportunity in Higher Education.

[ref20] Frijda N. H. Kuipers P. Terschure E. (1989). Relations between emotion, appraisal and emotional action readiness. J. Pers. Soc. Psychol. 57, 212–228. doi: 10.1037/0022-3514.57.2.212

[ref21] Gaskin J. (2016). “Group differences” stats tools package. Available at: http://statwiki.kolobkreations.com/

[ref22] Gonzaga G. C. Campos B. Bradbury T. (2007). Similarity, convergence, and relationship satisfaction in dating and married couples. J. Pers. Soc. Psychol. 93, 34–48. doi: 10.1037/0022-3514.93.1.34 17605587

[ref9003] Hatfield E. Cacioppo J. T. Rapson R. L. (1994). Emotional contagion. Cambridge University Press;Editions de la Maison des Sciences de l’Homme, Paris, France.

[ref23] Horn L. Nuñez A. M. (2000). Mapping the road to college: First-generation students' math track, planning strategies, and context of support. Washington, DC: Diane Publishing.

[ref24] Ickes W. (1993). Empathic accuracy. J. Pers. 61, 587–610. doi: 10.1111/j.1467-6494.1993.tb00783.x

[ref25] Ickes W. Hodges S. D. (2013). “Empathic accuracy in close relationships” in The Oxford handbook of close relationships. ed. Campbell J. A. S. L. (Oxford: Oxford University Press)

[ref26] Kim Y. K. Sax L. J. (2009). Student–faculty interaction in research universities: differences by student gender, race, social class, and first-generation status. Res. High. Educ. 50, 437–459. doi: 10.1007/s11162-009-9127-x

[ref27] Kitayama S. Mesquita B. Karasawa M. (2006). Cultural affordances and emotional experience: socially engaging and disengaging emotions in Japan and the United States. J. Pers. Soc. Psychol. 91, 890–903. doi: 10.1037/0022-3514.91.5.890 17059308

[ref28] Kline T. J. (2005). Psychological testing: a practical approach to design and evaluation. Thousand Oaks, CA: SAGE Publications.

[ref29] Kraus M. W. Stephens N. M. (2012). A road map for an emerging psychology of social class. Soc. Personal. Psychol. Compass 6, 642–656.

[ref30] Locke K. D. Horowitz L. M. (1990). Satisfaction in interpersonal interactions as a function of similarity in level of dysphoria. J. Pers. Soc. Psychol. 58, 823–831. doi: 10.1037/0022-3514.58.5.823 2348370

[ref9005] Lawrie S. L. Carter D. Nylund-Gibson K. Kim H. S. (2023). A tale of two belongings: Social and academic belonging differentially shape academic and psychological outcomes among university students [Unpublished manuscript]. 10.3389/fpsyg.2024.1394588 PMC11861437 40013255

[ref31] Markus H. R. Conner A. (2013). Clash! 8 cultural conflicts that make us who we are. Psychol. Sci. 20, 444–446.

[ref32] Matsumoto D. Yoo S. H. Fontaine J. (2008). Mapping expressive differences around the world: the relationship between emotional display rules and individualism versus collectivism. J. Cross-Cult. Psychol. 39, 55–74.

[ref33] Mayer J. D. Salovey P. (1997). “What is emotional intelligence?” in Emotional development and emotional intelligence: Educational implications. eds. Salovey P. Sluyter D. J. (New York City, NY: Basic Books), 3–34.

[ref34] Mayer J. D. Salovey P. Caruso D. R. (2002). Mayer-Salovey-Caruso emotional intelligence test (MSCEIT) item booklet, Toronto, Canada: MHS Assessments.

[ref35] Mesquita B. (2001). Emotions in collectivist and individualist contexts. J. Pers. Soc. Psychol. 80, 68–74. doi: 10.1037//0022-3514.80.1.6 11195892

[ref36] Mesquita B. Janxin L. (2007). “The cultural psychology of emotions” in Handbook of cultural psychology. eds. Kitayama S. Cohen D. (New York City, NY: The Guilford Press), 734–759.

[ref37] Mesquita B. Karasawa M. (2002). Different emotional lives. Cognit. Emot. 16, 127–141. doi: 10.1080/0269993014000176

[ref38] Murff S. H. (2005). The impact of stress on academic success in college students. ABNF J. 16, 102–104. 16268204

[ref39] Pascarella E. T. Pierson C. T. Wolniak G. C. Terenzini P. T. (2004). First-generation college students: additional evidence on college experiences and outcomes. J. High. Educ. 75, 249–284.

[ref40] Pool L. D. Qualter P. (2012). Improving emotional intelligence and emotional self-efficacy through a teaching intervention for university students. Learn. Individ. Differ. 22, 306–312. doi: 10.1016/j.lindif.2012.01.010

[ref41] Preston S. D. de Waal F. B. M. (2002). Empathy: its ultimate and proximate bases. Behav. Brain Sci. 25, 1–20. doi: 10.1017/S0140525X02000018 12625087

[ref42] Pryor J. H. Hurtado S. Saenz V. B. Santos J. L. Korn W. S. (2007). The American freshman: Forty year trends. Los Angeles: Higher Education Research Institute; University of California, Los Angeles.

[ref43] Reardon S. F. (2011). “The widening academic achievement gap between the rich and the poor: new evidence and possible explanations” in Whither opportunity?: Rising inequality, schools, and children's life chances. eds. Duncan G. J. Murnane R. J. (New York, NY: Russell Sage Foundation), 91–116.

[ref44] Rubin M. (2012). Social class differences in social integration among students in higher education: a meta-analysis and recommendations for future research. J. Divers. High. Educ. 5, 22–38.

[ref45] Rubin M. Evans O. McGuffog R. (2019). “Social class differences in social integration at university: implications for academic outcomes and mental health” in The social psychology of inequality. eds. Jetten J. Peters K. (Cham, Switzerland: Springer), 87–102.

[ref46] Savani K. Alvarez A. Mesquita B. Markus H. R. (2013). Feeling close and doing well: the prevalence and motivational effects of interpersonally engaging emotions in Mexican and European American cultural contexts. Int. J. Psychol. 48, 682–694. 22731253 10.1080/00207594.2012.688131

[ref47] Simpson J. A. Fletcher G. J. O. Campbell L. (2001). “The structure and function of ideal standards in close relationships” in Blackwell handbook of social psychology: Interpersonal processes. eds. Fletcher G. J. O. Clark M. S. (Oxford, UK: Blackwell Publishers), 86–106.

[ref48] Sirin S. R. (2005). Socioeconomic status and academic achievement: a meta-analytic of research. Rev. Educ. Res. 75, 417–453.

[ref49] Solomon R. C. (2004). Thinking about feeling: Contemporary philosophers on emotions, Oxford, UK: Oxford University Press.

[ref9004] Steiger J. H. (1990). Structural model evaluation and modification: An interval estimation approach. Multivariate behavioral research. 25, 173–180. doi: 10.1207/s15327906mbr2502_4 26794479

[ref50] Stephens N. M. Fryberg S. A. Markus H. R. Johnson C. S. Covarrubias R. (2012a). Unseen disadvantage: how American universities' focus on independence undermines the academic performance of first-generation college students. J. Pers. Soc. Psychol. 102, 1178–1197. doi: 10.1037/a0027143 22390227

[ref51] Stephens N. M. Townsend S. S. Markus H. R. Phillips L. T. (2012b). A cultural mismatch: independent cultural norms produce greater increases in cortisol and more negative emotions among first-generation college students. J. Exp. Soc. Psychol. 48, 1389–1393. doi: 10.1016/j.jesp.2012.07.008

[ref1001] Stephens N. M. Markus H. R. Phillips L. T. (2014). Social class culture cycles: How three gateway contexts shape selves and fuel inequality. Annual Review of Psychology, 65, 611–634. 10.1146/annurev-psych-010213-115143 24079532

[ref52] The World Bank. (2023). GINI index. Available at: https://data.worldbank.org/indicator/SI.POV.GINI

[ref53] Townsend S. S. Kim H. S. Mesquita B. (2014). Are you feeling what I’m feeling? Emotional similarity buffers stress. Soc. Psychol. Personal. Sci. 5, 526–533. doi: 10.1177/1948550613511

[ref54] Tsai J. L. Clobert M. (2019). “Cultural influences on emotion: established patterns and emerging trends” in Handbook of cultural psychology. eds. Cohen D. Kitayama S.. 2nd ed (New York City, NY: The Guilford Press).

[ref55] Uchida Y. Kitayama S. (2009). Happiness and unhappiness in east and west: themes and variations. Emotion 9, 441–456. doi: 10.1037/a0015634 19653765

[ref56] Vasquez-Salgado Y. Greenfield P. M. Burgos-Cienfuegos R. (2015). Exploring home-school value conflicts: implications for academic achievement and well-being among Latino first-generation college students. J. Adolesc. Res. 30, 271–305. doi: 10.1177/0743558414561297

[ref57] Verhofstadt L. L. Buysse A. Ickes W. Davis M. Devoldre I. (2008). Support provision in marriage: the role of emotional similarity and empathic accuracy. Emotion 8, 792–802. doi: 10.1037/a0013976 19102590

[ref58] Walton G. M. Cohen G. L. (2007). A question of belonging: race, social fit, and achievement. J. Pers. Soc. Psychol. 92, 82–96. doi: 10.1037/0022-3514.92.1.82 17201544

[ref59] Wang Q. (2004). The emergence of cultural self-constructs: autobiographical memory and self-description in European American and Chinese children. Dev. Psychol. 40, 3–15. doi: 10.1037/0012-1649.40.1.3 14700460

[ref60] Wilbur T. G. Roscigno V. J. (2016). First-generation disadvantage and college enrollment/completion. Socius 2, 67–90. doi: 10.1177/2378023116664351

[ref61] World Economic Forum. (2020). The global social mobility report 2020: Equality, opportunity and a new economic imperative. Available at: https://www.weforum.org/publications/global-social-mobility-index-2020-why-economies-benefit-from-fixing-inequality/

