# Supplementary material for: The role of emotional similarity and emotional accuracy in belonging and stress among first-generation and continuing-generation students
Source: Front Psychol. 2024 Feb 14;15:1355526. doi: 10.3389/fpsyg.2024.1355526 (PMC10899461; doi:10.3389/fpsyg.2024.1355526)
Supplement: Supplementary file 1 [file Data_Sheet_1.PDF]

Supplementary Materials

Table S1

Moderation Analysis: Differences in the Paths of the Model Between the Two Groups

Path in the model	Sample				
	First Generation		Continuing Generation		
	Estimate	P	Estimate	P	z-score
NE Emotional Similarity→Social Belonging	-0.359	0.126	-0.859	0.006	-1.283
NE Emotional Accuracy→ Social Belonging	0.313	0.225	0.657	0.025	0.881
NE Emotional Similarity →Stress	0.251	0.165	0.557	0.005	1.145
ND Emotional Accuracy→Stress	-0.737	0.006	-0.146	0.606	1.519
Social Belonging→Stress	-0.113	0.143	-0.293	0.000	-1.744*
ND Emotional Similarity →Stress	1.153	0.000	0.743	0.003	-1.037

Note: NE – Negative Engaged, ND – Negative Disengaged

*p-value < 0.10. **p-value < 0.05. ***p-value < 0.01.

The "Group Differences" tool in AMOS (Gaskin, 2016) was used. To test whether our model was invariant across college generational status, the paths of the original model were constrained to be equal across the two groups and the restricted and unrestricted models were compared. The change in fit indices were examined to see whether the added constraints affected fit. Follow-up invariance tests showed that two structural covariances needed to vary freely. Negative Disengaged Emotional Similarity and Negative Disengaged Emotional Accuracy were left to freely vary. This new model presented no structural differences in paths between first- and continuing-generation students, with the exception of Belonging In other words, the overall model structure for the amended model is almost identical for first- and continuing-generation students (See Figures S1 and S2).

Figure S1

Structural Equation Model (SEM) for Testing the Mediation of Belonging on Stress for First-Generation Students

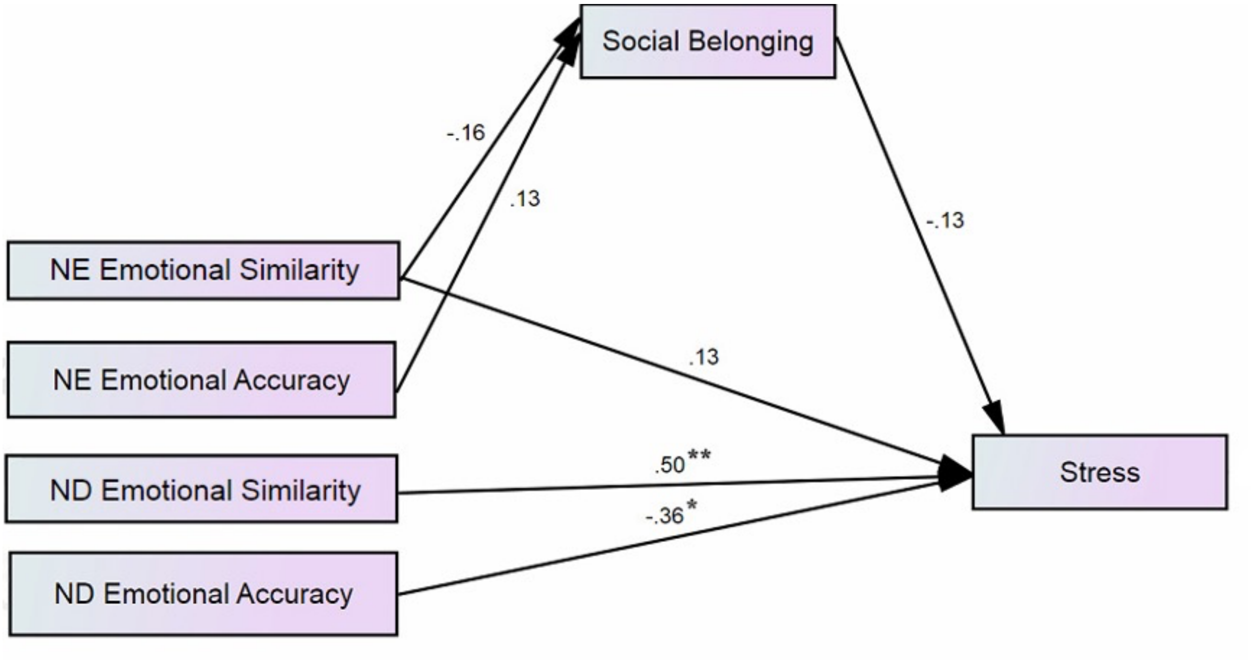

Note. *- p <.05, **- p <.01.

Figure S2

Structural Equation Model (SEM) for Testing the Mediation of Belonging on Stress for Continuing-Generation Students

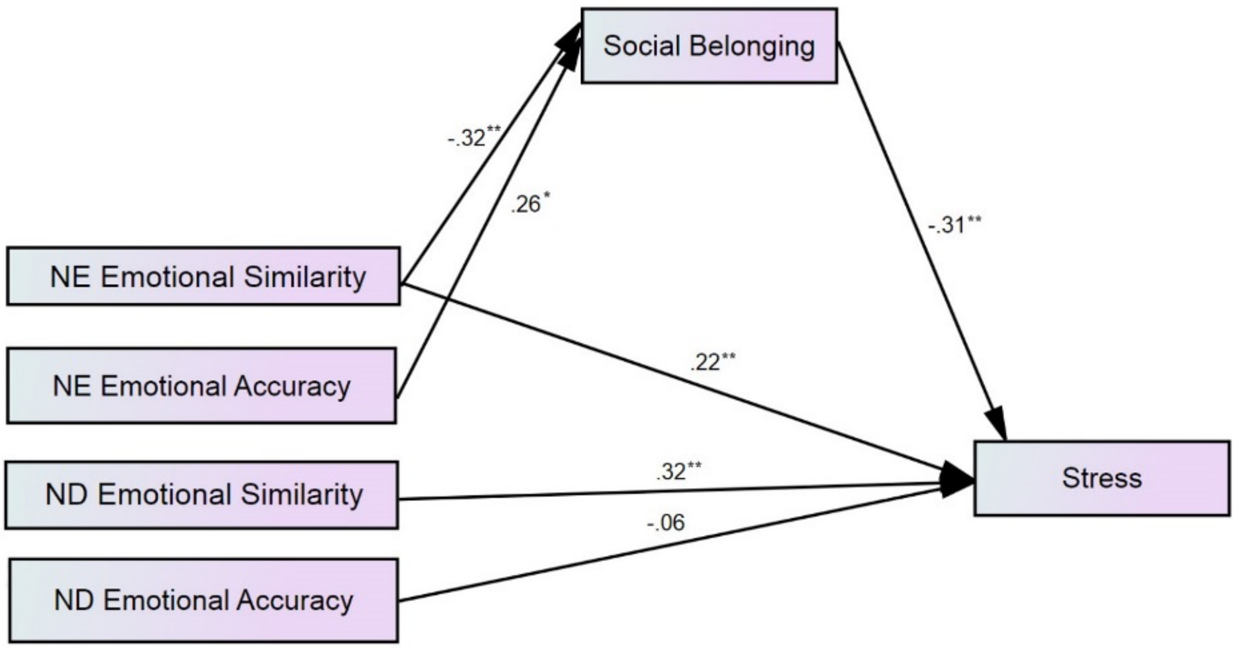

Note. *- p < .05, **- p <.01.
